# Toward a Unified Nomenclature System for Highly Pathogenic Avian Influenza Virus (H5N1)

**DOI:** 10.3201/eid1407.071681

**Published:** 2008-07

**Authors:** WHO, OIE, FAO H, N, Evolution Working Group

**Keywords:** Highly pathogenic avian influenza, H5N1, molecular epidemiology, viral evolution, phylogenetics, nomenclature, online report

Highly pathogenic avian influenza (HPAI) virus (H5N1) has appeared in >60 countries and continues to evolve and diversify at a concerning rate. Because different names have been used to describe emerging lineages of the virus, this study describes a unified nomenclature system to facilitate discussion and comparison of subtype H5N1 lineages.

The continuing geographic expansion and rapid evolution of HPAI subtype H5N1 virus across 3 continents is hindering control and eradication efforts in affected countries and raising public health concerns about a potential influenza pandemic. Since 1997, when the virus was discovered to cause disease and death in humans in Hong Kong, researchers have monitored the movement of the virus from region to region. Its molecular evolution has been characterized to better understand the spread of the virus and thus help prevent its perpetuation in poultry populations. Specific mutations and reassortment events that may enhance the virus’s ability to infect and be transmitted to humans (*1*–*7*) have also been scrutinized. Therefore, much effort has been spent to delineate the emerging lineages of the HPAI viruses (H5N1) from their earliest known progenitor, A/goose/Guangdong/96 (Gs/GD). From this ancestral virus, numerous lineages have evolved and because of rapid transcontinental spread, numerous publications have used different names to classify similar (if not identical) groups of viruses within the Gs/GD-like lineage (*1*–*6*). As a result, discussion and comparison of virus isolates have been hindered by a lack of uniformity in nomenclature, often leading to confusion in the interpretation of research results. The now routine practice of genome sequencing has also dramatically increased the sequence information available for analyses, adding to the complexity of examining the evolutionary relationships among HPAI virus (H5N1) isolates.

To address these issues, an international group of scientists and collaborators, referred to as the H5N1 Evolution Working Group, was convened at the Options for the Control of Influenza VI Conference in June 2007 in Toronto, Ontario, Canada. Their goal was to develop a unified nomenclature system for the classification of HPAI viruses (H5N1) belonging to the Gs/GD-like virus lineage. The initiative, which was encouraged and approved by 3 international agencies (the World Health Organization [WHO]), the World Organisation for Animal Health [OIE], and the Food and Agriculture Organization [FAO]), set out to unify the nomenclature system to simplify interpretation of sequence and surveillance data from different laboratories and to remove stigmatizing labeling of HPAI virus (H5N1) clades by geographic reference. Although most genes of the HPAI virus (H5N1) genome have undergone reassortment leading to their replacement by genes from lineages distinct from Gs/GD, the hemagglutinin (HA) protein gene has not been replaced since its emergence in 1996 (*1*). Therefore, monitoring the evolution of the Gs/GD HA lineage provides an initial constant by which H5N1 strains may be effectively compared. Taking these factors into account, we performed phylogenetic analyses on all of the publicly available subtype H5N1 HA sequences that have evolved in the Gs/GD lineage and designed a classification system. The results support the concept that the HPAI viruses (H5N1) currently circulating can be effectively grouped into multiple clades, herein designated by a hierarchical numbering system. Global adoption of the proposed H5 clade nomenclature and its expansion to other influenza lineages and genes, including other animal influenza virus subtypes, will benefit human and animal influenza research and public health.

## The Study

**Figure 1:**
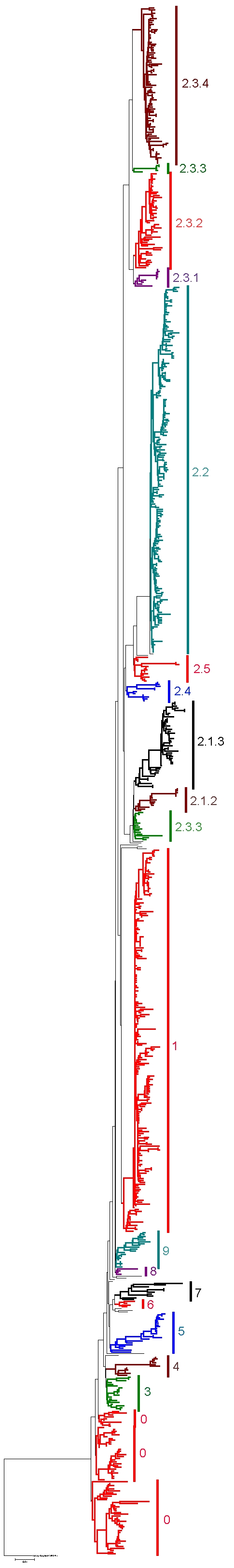
Neighbor-joining tree of 859 H5N1 isolates constructed by using PAUP* version 4.0b10 (*9*) with 1,000 bootstrap replicates. The tree was rooted by using the highly pathogenic avian influenza virus (H5N1) strain A/turkey/England/50–92/91, a historical European H5N1 virus closely related to the Gs/GD lineage (*10*). Clades are color coded with isolates not given a clade designation in light green. Maximum-likelihood trees used for comparison were constructed by using GARLI version 0.951 (*11*). Bayesian analysis for comparison was conducted with MrBayes version 3.1 (*12*) by using 4 replicates of 2 million generations, sampled every 100 generations, with 6 chains. The convergence of Markov Chain Monte Carlo chains was confirmed for each dataset by using Tracer version 1.3 (http://tree.bio.ed.ac.uk/software/tracer). Estimates of the statistical significance of phylogenies were calculated by performing 1,000 NJ bootstrap replicates, and Bayesian posterior probabilities were calculated from the consensus of 60,000 trees after excluding the first 20,000 trees (25%) as burn-in. Scale bar represents 0.01-nt changes.

**Figure 2:**
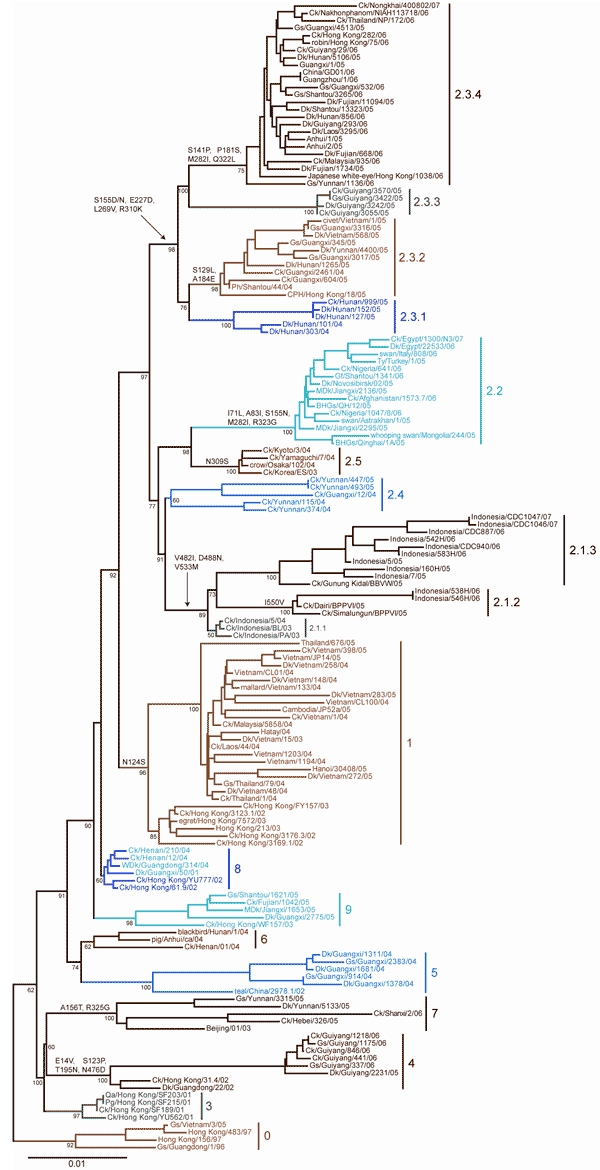
Neighbor-joining tree of 158 H5N1 isolates constructed by using PAUP* version 4.0b10 (*9*). Estimates of the phylogenies were calculated by performing 1,000 neighbor-joining bootstrap replicates. Distinct amino acid residues shared only by isolates within a particular clade are shown on the line above the clade-defining node when present. Amino acid substitutions represent change relative to Gs/GD/1/96. The small tree was rooted at the clade 0 node for larger scaling. Scale bar represents 0.01-nt changes. Ck, chicken; Gs, goose; Dk, duck; Ph, pheasant; CPH, Chinese pond heron; Ty, turkey; Gf, Guinea fowl; MDk, migratory duck; BHGs, bar-headed goose; WDk, wild duck; Qa, quail; Pg, pigeon.

Nucleotide sequences of the HA gene of HPAI viruses (H5N1) were collected from publicly available databases: GenBank National Center for Biotechnology (NCBI) and the Influenza Sequence Database of Los Alamos National Laboratories (LANL). The analysis only included nearly full-length HA sequences (i.e., at least 1,600 nt in length) to ensure robust statistical support (Table 1). Multiple sequence alignment of 871 HA sequences was performed with ClustalW (www.ebi.ac.uk/Tools/clustalw2). The final alignment length was 1,707 nt. Isolates with 100% sequence similarity (i.e., redundant sequences) were identified and removed, giving a final alignment of 859 sequences. The appropriate DNA substitution model and γ-rate heterogeneity were determined with MrModeltest v2.2 (*8*) and used in all subsequent analyses. The neighbor-joining (NJ), maximum-likelihood (ML), and Bayesian methods used to construct trees for comparison are detailed in the legend of Figure 1. For ease of display, and also to ensure that the clade topology would be maintained if fewer isolates were used, a smaller dataset of 158 subtype H5N1 HA sequences was analyzed that included representative vaccine strains, reference serum strains, many human isolates, pathogenesis study strains, and geographically diverse isolates (Figure 2). Phylogenetic analyses were conducted on this dataset as described for Figure 1.

**Table 1:** Criteria used for clade designation

No.	Criteria
1	Maintain previously designated clade numbers where possible (i.e., clade 2.2 remains 2.2 and clade 1 remains 1)
2	New clade designations based on phylogenetic tree topology derived from all available sequences (the large tree)
	H5N1 progenitors (closest to Gs/Guangdong/1/96) re-designated as clade 0
	Subsequent clades numbered starting from clade 3 (i.e., clades 3–9)
	Clades designated by presence of a distinct common node shared by at least 4 isolates (in a monophyletic group)
	Additional branches designated as a single clade evolves into more than one distinct lineage (i.e., clade 2.2 or clade 2.3.1; based on sharing of a common node and monophyletic grouping)
3	Average percentage pairwise distances between and within clades (using Kimura 2-parameter)
	Distinct clades should have >1.5% average distances between other clades
	Distinct clades should have <1.5% average distances within the clade (may be slightly higher in clades with highly evolved outliers; i.e., Ck/Shanxi/2/2006 in clade 7)
4	Bootstrap (based on 1,000 neighbor-joining bootstrap replicates) >60% bootstrap value at clade-defining node

To quantify the nucleotide distances between and within groups identified on the phylogenetic tree, the average pairwise distances (between and within clades) were calculated by using MEGA version 3.1 (www.flu.org.cn/en/download-51.html) (*13*) by the NJ method with the Kimura 2-parameter model. Each distinct clade was determined to have an average distance >1.5% from other clades and an average distance <1.5% within the clade. Certain clades that comprise highly evolved HA genes depart slightly from these criteria; higher average intraclade distances were observed (i.e., Ck/Shanxi/2/2006 in clade 7).

Clade assignments were made by following several criteria used collectively to rationally name groups by a clade number. The criteria used to define clades are described in Table 1. Using these specific criteria, we identified and numbered 10 unique clades from the consensus topology of the large trees generated (Figure 1). The clade designations were then confirmed by the consensus topology of the smaller trees generated (Figures 1, 2). The topology of each clade was almost identical, and major clades were identified with consistency by using any of the 3 phylogenetic tree reconstruction methods (NJ, ML, and Bayesian). Also, the identified clades were consistent between the large and small datasets. Although the overall topologic organization between the large and small tree varied slightly (i.e., the positions of clades 7 and 8 are changed), the monophyletic grouping and bootstrap support for each clade remained predominately unchanged. However, trees derived from small datasets often yielded minor discrepancies; e.g., 4 isolates that were designated as clade 9 in the large tree were grouped with clade 8 in the smaller tree. The discrepancies in grouping between the large and small trees indicate the importance of using the largest datasets possible when classifying viral sublineages by phylogenetic analyses. Several of the identified clades were found to have distinct amino acid residues shared by members of that clade. To identify clade-specific amino acid residues, amino acid alignments were constructed for each clade, and residues shared by all members of that clade were compared with the Gs/GD/1/96 virus. Distinct shared amino acid residues are shown for each clade at the clade-defining node in Figure 2.

## Conclusions

Using the clade designation criteria proposed in Table 1, this study has identified 10 unique first-order numbered clades of the HPAI viruses (H5N1) in the Gs/GD-like lineage (clades 0–9). The group of HA genes previously designated as clade 2 showed a level of diversity that far exceeds the current definition of a clade; therefore, this group was also separated into 5 additional second-order clades (clades 2.1–2.5). Clades 2.1 (avian/human isolates from Indonesia) and 2.3 (avian/human isolates from the People’s Republic of China; Hong Kong; Vietnam; Thailand; Lao People’s Democratic Republic; and Malaysia) were also further delineated into third-order groups (clades 2.1.1–2.1.3 and 2.3.1–2.3.4), respectively. The origins of isolates belonging to each clade are described in Table 2. For each clade identified, a representative prototype virus is listed to facilitate interpretation of the proposed numbering system (Table 2). As other studies have shown, the primary clade defining factor appears to be spatio-temporal because most distinct clades consist of isolates within close geographic proximity to one another or from specific time periods (perhaps as a result of heightened transmission during outbreak periods) (*2*–*7*). Notably, clade 2.2 comprises isolates from more widespread geographic areas (3 continents), which is likely to reflect movement of the virus through long-distance spread as a result of poultry trade or wild bird migration (*2*,*3*,*6*,*7*).

**Table 2:** Clade descriptions with isolation period, source, and geographic location*

Clade	Year	Geographic location	Isolation source	Description and strain name
0	1996–2002	PRC, Hong Kong	Avian/human	Early progenitors of H5N1; HK/PRC 1997 avian influenza outbreak **Gs/Guangdong/1/96**
3	2000–2001	PRC, Hong Kong, Vietnam	Avian	**Ck/Hong Kong/YU562/2001**
4	2002/2003	PRC, Hong Kong	Avian	**Gs/Guiyang/337/2006**
	2005/2006	Guiyang, PRC	Avian	Described as Guiyang 1 (*5*)
5	2000–2003	PRC, Vietnam	Avian	**Gs/Guangxi/914/2004**
	2004	Guangxi, PRC	Avian	
6	2002/2004	PRC	Avian	**Ck/Hunan/01/2004**
7	2002/2004	PRC	Avian/human	Human case from Beijing in 2003
	2005/2006	Yunnan, Hubei, and Shanxi, PRC	Avian	Described as Yunnan 2 (*5*) **Ck/Shanxi/2/2006**
8	2001–2004	Hong Kong, PRC	Avian	**Ck/Hong Kong/YU777/2002**
9	2003–2005	PRC	Avian	**Dk/Guangxi/2775/2005**
1	2002/2003	Hong Kong, PRC	Avian/human	Described as Guangdong (*3*)
		Vietnam, Cambodia, Thailand, Laos, Malaysia	Avian/human	Spread of H5N1 to southeast Asia; described as Vietnam/Thailand/Malaysia (*3*) **Vietnam/1203/2004**
2.1.1	2003–2005	Eastern Indonesia	Avian	Described as Indonesia (ref 3) **Ck/Indonesia/BL/2003**
2.1.2	2005–2006	Western Indonesia	Avian/human	Primarily avian with human cluster from Medan; described as Indonesia (*3*) **Indonesia/538H/2006**
2.1.3	2004–2007	Eastern and western Indonesia	Avian/human	Described as Indonesia (*3*) **Indonesia/5/2005**
2.2	2005	Qinghai Lake, Jiangxi, PRC	Avian	Progenitors from Qinghai Lake outbreak; described as Qinghai-like (*5*)
	2005–2007	Mongolia, Europe, Middle East, Africa	Avian/human	Long-distance spread of H5N1; described as EMA clade (*4*) **BHGs/Qinghai/1A/2005**
2.3.1	2003–2005	Hunan and Guangdong, PRC	Avian	Described as Hunan (*3*) **Dk/Hunan/303/2004**
2.3.2	2004–2006	Hong Kong, southern PRC	Avian	Described as Mixed/Vietnam 2 (*3*,*5*)
	2005	Vietnam	Avian	Described as Mixed/Vietnam 2 (*3*,*5*) **Ck/Guangxi/2461/2004**
2.3.3	2004	Hunan, PRC	Avian	**Ck/Guiyang/3055/2005**
	2005	Guiyang, PRC	Avian	Described as Guiyang 2 (*5*)
2.3.4	2005–2006	Hong Kong, PRC, Thailand, Laos, Malaysia	Avian/human	Described as Fujian-like (*5*) **Dk/Fujian/1734/2005**
2.4	2002–2005	PRC (predominantly Yunnan and Guangxi)	Avian	Described as Yunnan (*3*) **Ck/Yunnan/115/2004**
2.5	2003/2004	PRC, Korea, Japan	Avian	Spread of H5N1 to east Asian countries
	2006	Shantou, PRC	Avian	Described as Guangdong/2006 (*5*) **Ck/Korea/ES/2003**

*PRC, People’s Republic of China; BHGs, bar-headed goose; Ck, chicken; Dk, duck; Gs, goose. Strain name in **boldface** represents the prototype virus for that clade.

The evolution of the H5 HA in avian hosts shows a notable difference from the typical evolution of HA genes from human influenza viruses. The evolution of the H3 HA since 1968 is characterized by a limited diversity among circulating strains. This lack of diversity is clearly the consequence of rapid extinction after the emergence of new clades and lineages. As expected, the evolutionary tree of human influenza HA genes has extended trunks and extremely short branches (*14*,*15*). In contrast, multiple avian influenza A HA clades continue to evolve and co-circulate in different regions and species; hence, the unprecedented need for a nomenclature system that has been unnecessary for human influenza genes.

The results from this study indicate that the HPAI H5N1 viruses can be grouped into several clades designated by a numbering system that can continue to be expanded as these viruses continue to evolve. By establishing this nomenclature system and guidelines for naming clades, this information can be used in the future as criteria for assigning new clades as new lineages of HPAI H5N1 variants emerge.

## References

[1] Duan L, Campitelli L, Fan XH, Leung YH, Vijaykrishna D, Zhang JX, Characterization of low-pathogenic H5 subtype influenza viruses from Eurasia: implications for the origin of highly pathogenic H5N1 viruses. J Virol. 2007;81:7529–39. 10.1128/JVI.00327-07 17507485 PMC1933357

[2] Ducatez MF, Olinger CM, Owoade AA, Tarnagda Z, Tahita MC, Sow A, Molecular and antigenic evolution and geographical spread of H5N1 highly pathogenic avian influenza viruses in western Africa. J Gen Virol. 2007;88:2297–306. 10.1099/vir.0.82939-0 17622635

[3] Chen H, Smith GJD, Li KS, Wang J, Fan XH, Rayner JM, Establishment of multiple sublineages of H5N1 influenza virus in Asia: Implications for pandemic control. Proc Natl Acad Sci U S A. 2006;103:2845–50. 10.1073/pnas.0511120103 16473931 PMC1413830

[4] Salzberg SL, Kingsford C, Cattoli G, Spiro DJ, Janies DA, Aly MM, Genome analysis linking recent European and African influenza (H5N1) viruses. Emerg Infect Dis. 2007;13:713–8. 17553249 10.3201/eid1305.070013 PMC2432181

[5] Smith GJD, Fan XH, Wang J, Li KS, Qin K, Zhang JX, Emergence and predominance of an H5N1 influenza variant in China. Proc Natl Acad Sci U S A. 2006;103:16936–41. 10.1073/pnas.0608157103 17075062 PMC1636557

[6] Smith GJD, Naipospos TSP, Nguyen TD, de Jong MD, Vijaykrishna D, Usman TB, Evolution and adaptation of H5N1 influenza virus in avian and human hosts in Indonesia and Vietnam. Virology. 2006;350:258–68. 10.1016/j.virol.2006.03.048 16713612

[7] Wallace RG, Hodac H, Lathrop RH, Fitch WM. A statistical phylogeography of influenza A H5N1. Proc Natl Acad Sci U S A. 2007;104:4473–8. 10.1073/pnas.0700435104 17360548 PMC1838625

[8] Nylander JAA. MRMODELTEST 2. Evolutionary Biology Centre, Uppsala University, Uppsala, Sweden; 2004 [cited 2007 Mar 12]. Available from http://www.abc.se/~nylander

[9] Swofford DL. PAUP: phylogenetic analysis using parsimony, version 4. Sunderland (MA): Sinauer Academic Publishers; 2001.

[10] Wood GW, Banks J, McCauley JW, Alexander DJ. Deduced amino acid sequences of the haemagglutinin of H5N1 avian influenza virus isolates from an outbreak in turkeys in Norfolk, England. Arch Virol. 1994;134:185–94. 10.1007/BF01379117 7506519

[11] Zwickl D. Genetic algorithm approaches for the phylogenetic analysis of large biological sequence datasets under the maximum likelihood criterion. PhD thesis, University of Texas at Austin; 2006 [cited 2008 Jun 5]. Available from http://www.bio.utexas.edu/faculty/antisense/garli/Garli.html

[12] Huelsenbeck JP, Ronquist FR. MRBAYES: Bayesian inference of phylogenetic trees. Bioinformatics. 2001;17:754–5. 10.1093/bioinformatics/17.8.754 11524383

[13] Kumar S, Tamura K, Nei M. MEGA3: an integrated software for Molecular Evolutionary Genetics Analysis and sequence alignment. Brief Bioinform. 2004;5:150–63. 10.1093/bib/5.2.150 15260895

[14] Fitch WM, Leiter JM, Li XQ, Palese P. Positive Darwinian evolution in human influenza A viruses. Proc Natl Acad Sci U S A. 1991;88:4270–4. 10.1073/pnas.88.10.4270 1840695 PMC51640

[15] Wolf YI, Viboud C, Holmes EC, Koonin EV, Lipman DJ. Long intervals of stasis punctuated by bursts of positive selection in the seasonal evolution of influenza A virus. Biol Direct. 2006;1:34. 10.1186/1745-6150-1-34 17067369 PMC1647279

